# Hyperinsulinemia-induced microglial mitochondrial dynamic and metabolic alterations lead to neuroinflammation *in vivo* and *in vitro*

**DOI:** 10.3389/fnins.2022.1036872

**Published:** 2022-11-16

**Authors:** Xiaohan Yang, Yuan Xu, Wenting Gao, Li Wang, Xinnan Zhao, Gang Liu, Kai Fan, Shuang Liu, Huimin Hao, Siyan Qu, Renhou Dong, Xiaokai Ma, Jianmei Ma

**Affiliations:** ^1^Department of Anatomy, College of Basic Medical Sciences, Dalian Medical University, Dalian, China; ^2^Department of Morphology, College of Basic Medical Sciences, Dalian Medical University, Dalian, China; ^3^Institute of Genome Engineered Animal Models for Human Disease, National Center of Genetically Engineered Animal Models, College of Integrative Medicine, Dalian Medical University, Dalian, China; ^4^College of Medical Imaging Laboratory and Rehabilitation, Xiangnan University, Chenzhou, China; ^5^Key Laboratory of Medical Imaging and Artificial Intelligence of Hunan Province, Xiangnan University, Chenzhou, China; ^6^National-Local Joint Engineering Research Center for Drug-Research and Development (R&D) of Neurodegenerative Diseases, Dalian Medical University, Dalian, China; ^7^The Second Affiliated Hospital of Guangzhou Medical University, Guangzhou, China

**Keywords:** hyperinsulinemia, insulin resistance, neuroinflammation, Alzheimer’s disease, microglia

## Abstract

Numerous studies have demonstrated that type 2 diabetes (T2D) is closely linked to the occurrence of Alzheimer’s disease (AD). Nevertheless, the underlying mechanisms for this association are still unknown. Insulin resistance (IR) hallmarked by hyperinsulinemia, as the earliest and longest-lasting pathological change in T2D, might play an important role in AD. Since hyperinsulinemia has an independent contribution to related disease progressions by promoting inflammation in the peripheral system, we hypothesized that hyperinsulinemia might have an effect on microglia which plays a crucial role in neuroinflammation of AD. In the present study, we fed 4-week-old male C57BL/6 mice with a high-fat diet (HFD) for 12 weeks to establish IR model, and the mice treated with standard diet (SD) were used as control. HFD led to obesity in mice with obvious glucose and lipid metabolism disorder, the higher insulin levels in both plasma and cerebrospinal fluid, and aberrant insulin signaling pathway in the whole brain. Meanwhile, IR mice appeared impairments of spatial learning and memory accompanied by neuroinflammation which was characterized by activated microglia and upregulated expression of pro-inflammatory factors in different brain regions. To clarify whether insulin contributes to microglial activation, we treated primary cultured microglia and BV2 cell lines with insulin *in vitro* to mimic hyperinsulinemia. We found that hyperinsulinemia not only increased microglial proliferation and promoted M1 polarization by enhancing the production of pro-inflammatory factors, but also impaired membrane translocation of glucose transporter 4 (GLUT4) serving as the insulin-responding glucose transporter in the processes of glucose up-taking, reduced ATP production and increased mitochondrial fission. Our study provides new perspectives and evidence for the mechanism underlying the association between T2D and AD.

## Introduction

Alzheimer’s disease (AD) is the most common cause of dementia (Bondi et al., 2017; Soria Lopez et al., 2019). Early studies have indicated that metabolic abnormalities occur in the relevant brain regions of AD patients (Rapoport et al., 1986; Heiss et al., 1989). And metabolic disturbances, particularly type 2 diabetes (T2D) increase the risk of cognitive impairment and AD (Stewart and Liolitsa, 1999; Pugazhenthi et al., 2017; Boccardi et al., 2019). However, the underlying mechanisms for this association are still unclear. Although the etiology and process of T2D are complex, insulin resistance (IR) is the earliest and longest-lasting pathophysiological change (Groop, 1999; Lebovitz, 2001). IR is defined as an impaired biologic response to insulin stimulation of target tissues resulting in a compensatory increase in insulin production and hyperinsulinemia (Shanik et al., 2008; Freeman and Pennings, 2022). IR can result in several metabolic consequences such as hyperglycemia, hypertension, dyslipidemia, visceral adiposity, and so on (Shanik et al., 2008; Yazıcı and Sezer, 2017; Beverly and Budoff, 2020). Among them, hyperinsulinemia has an independent contribution to related disease progressions (Hegde et al., 2019; Zhang et al., 2021). For instance, hyperinsulinemia can activate mononuclear macrophages toward “M1-like” state to release a large amount of pro-inflammatory factors in the periphery (Manowsky et al., 2016; Kubota et al., 2018), suggesting hyperinsulinemia as an independent factor might be involved in inflammation.

Although few, some studies have shown that insulin plays multi-functions in central nervous system (CNS) (Banks et al., 2012; Kleinridders et al., 2014; Agrawal et al., 2021). Most insulin in the brain derives from circulating pancreatic insulin which enters the brain primarily across the blood–brain barrier (BBB) (Gray and Barrett, 2018). Insulin levels in cerebrospinal fluid (CSF) are much lower than that in plasma (Wallum et al., 1987; Schwartz et al., 1991). However, insulin receptors (InsR) are expressed on all cell types of brain and have various expression levels in the different brain regions, suggesting that insulin signaling has important and diverse roles (Pomytkin et al., 2018). The studies of insulin action in brain mainly focus on the states and functions of neurons, such as neurosynaptic regulation, neuronal polarization, and survival (Mielke and Wang, 2011; Grasso et al., 2013), but less on glial cells, especially microglia.

Microglia are the brain resident immune cells serving as an immunological surveillant, which can be rapidly activated to address the changes of microenvironment in the CNS, and therefore participate in pathological alterations of almost all CNS diseases including AD (Nayak et al., 2014; Colonna and Butovsky, 2017; Wolf et al., 2017). Extensive literatures demonstrate that neuroinflammation characterized by microglial activation and increased expression of inflammatory factors is the characteristic pathological change in the pathogenesis of AD (Hansen et al., 2018). Actually, there is a complex link between IR and neuroinflammation. IR-induced hyperglycemia and free fatty acids could cause and aggravate neuroinflammation *via* microglia activation (Van Dyken and Lacoste, 2018; Wanrooy et al., 2018; Wenzel et al., 2020; Yang et al., 2020). However, the effects of hyperinsulinemia on microglial activation in IR remain unclear. Therefore, to demonstrate the effect of hyperinsulinemia in early T2D on microglial activation and neuroinflammation would be helpful for clarifying the relationship between IR and AD pathogenesis.

Hyperinsulinemia is involved in alterations of cellular metabolism by impairing the intracellular transport of glucose and mitochondrial function (Lee et al., 2017; Turner et al., 2020). Meanwhile, altered metabolism by various environmental stimuli and cellular stresses is also an important factor affecting microglial functional status and polarization (Mehta et al., 2017; Lynch, 2020). Mitochondria as the energy-producing organelle has highly dynamic changes undergoing coordinated cycles of fission and fusion which mirror its function (Hatefi, 1993; Chan, 2020). Numerous studies have demonstrated that mitochondrial oxidative stress in microglia was involved in the development of AD, and elevated generation of reactive oxygen species (ROS) and loss of mitochondrial membrane potential through various mechanisms have been observed (Agrawal and Jha, 2020). However, the effects of hyperinsulinemia on mitochondrial dynamics and energy metabolism in microglia are poorly understood.

In the present study, we used high-fat-diet (HFD)-induced IR mouse model and combined with *in vitro* experiments. The series metabolic indices assays and key changes in the insulin signaling were used to determine the *in vivo* model success. The activation of microglia and the expression of pro-inflammatory factors in both periphery and the CNS were detected by quantitative real-time PCR (qRT-PCR), Western blot, immunohistochemistry, and cognitive function was analyzed by behavior analysis. Further, by *in vitro* study, the effects of hyperinsulinemia in primary microglia and BV2 cell line were determined after prolonged insulin treatment. The activation, proliferation, and active status, cellular glucose transporter 4 (GLUT4) expression, mitochondrial dynamics, and ATP production of microglia were examined. Our results showed that hyperinsulinemia decreased membranous expression of GLUT4, and impaired mitochondrial function indicated by increased phosphorylation of dynamin-related protein 1 (DRP1) at Ser616 and decreased Mitofusin 2 (MFN2) protein expression. All those suggested that hyperinsulinemia could lead to microglia M1 activation and proliferation by altered energy metabolism in IR condition.

## Materials and methods

### Animals

Male C57BL/6J mice (4 weeks old) purchased from specific pathogen free (SPF) animal central of Dalian Medical University. Mice were housed 5 per cage under a 12-h light-dark cycle at a stable temperature (24 ± 2°C), and unrestricted access to food and water. All experimental protocols were strictly performed according to the National Laboratory Animal Research Committee’s nursing guidelines, and the experimental animals were approved by the Ethics Committee of Dalian Medical University and the Animal Protection and Use Committee (No. AEE21103).

### Establishment of high-fat diet-induced insulin resistance mice model

The mice were randomly assigned standard diet (SD) and HFD feeding groups (*n* = 40). The initial body weight between the two groups has no significant difference. The SD mice were fed with standard diet, and the HFD mice were fed with high-fat diet containing 60% kcal fat (Research Diets 12492, NJ, United States) for 12 weeks. During the experiment period, body weight was examined every week. After 12 weeks, the mice were subjected to further behavioral tests or specimen collections.

### Morris water maze test

Morris water maze was used for the assessment of spatial learning and memory after mice had been fed by SD/HFD for 12 weeks. The MWM test was performed in a circular water tank with a diameter of 110 cm and a height of 60 cm. The tank was filled to a depth of 35 cm with clear, 24 ± 1°C water. A circular escape platform made of transparent Plexiglas (diameter: 10 cm) was submerged 1 cm below the surface of the water. Various brightly colored signs were placed around the maze. During all trials, the location of the hidden platform and signs were kept constant. The experimental environment was noise free and maintained at 25 ± 2°C. MWM was performed for six consecutive days, the mice were given single training trials for initial 5 days. Each day consisted of 4 swim trials (maximum 90 s per trial) with different quadrant starting positions for each trial. One by one mouse was released into the water maze at one of four quadrants with their heads facing the wall of the maze. The mice left for 90 s to find the invisible platform. Twenty-four hours after the last training, the mice were placed in the opposite quadrant of the platform and were allowed to swim for 60 s without a platform present. The whole process was recorded by a camera; the escape latency (time to reach the platform, in seconds), swimming speed, and time spent on target quadrant in the probe test were analyzed by a video tracking system with EthoVision version software.

### Measurements of fasting blood glucose and glucose tolerance tests

Mice were fasted overnight (16 h), tail tip blood was collected for the fasting blood glucose measurement. For GTT, before and after glucose (2 g/kg) (Nunez et al., 2009) intraperitoneal injection (i.p.) for 15, 30, 60, and 120 min, blood glucose concentration was measured with a fast blood glucose meter. Then the time (min) − glucose concentration (mmol/L) curve was drawn and the area under the curve (AUC) was calculated.

### Measurement of serum lipid concentration of mice

Blood samples from mice were collected *via* cardiac puncture. After clotting at room temperature for 3 h, blood samples were centrifuged at 3000 rpm/min for 15 min. Serum samples were transferred to new tubes and stored at -80°C. Serum total cholesterol (TC), triacylglycerol (TG), low-density lipoprotein (LDL), and high-density lipoprotein (HDL) concentrations were measured using enzymatic kits (Nanjing Jiancheng, Nanjing, China) according to the manufacturers’ protocols.

### Tissue section preparation and immunohistochemical staining

Mice were deeply anesthetized with tribromoethanol and perfused transcardially with phosphate buffered saline (PBS) followed by 4% paraformaldehyde perfusion. The brains were removed and fixed in 4% paraformaldehyde at 4°C for 24 h, then transferred to 20 and 30% sucrose solution in turn at 4°C for dehydration until it sank to the bottom of the tube. 18-μm sections of the brain were collected using a freezing microtome (Leica, Nussloch, Germany) and stored at -30°C. Immunohistochemistry (IHC) staining was conducted using the standard protocol as we described by previously (Zhao et al., 2021). The sections were incubated rabbit anti-ionized calcium binding adapter molecule 1 (Iba-1) polyclonal antibody (1:500, Wako, Saitama, Japan) at 4°C overnight. Images were captured by the Nikon digital camera system (DS-Fi1) in combination with microscopy (Nikon eclipse 80i, Nikon, Tokyo, Japan).

### Brain CD11b^+^ microglia isolation

Whole brains from SD- or HFD-feeding mice were dissociated to single cells suspensions using the Adult Brain Dissociation Kit (Miltenyi Biotec, Cologne, Germany), then microglia were further isolated by using cluster of differentiation 11b (CD11b) MicroBeads, MS Column and MiniMACS™ Separator (Miltenyi Biotec, Cologne, Germany), and pure microglia cells were used for subsequent experiments.

### Cell culture and treatments

As the description in our previous publications (Zhao et al., 2021), primary microglia were obtained from postnatal day 1 C57BL/6 mice, and BV2 cell lines were also used in the experiments. Both primary microglia and BV2 cells were cultured in Dulbecco’s modified Eagle’s medium (DMEM) containing 10% fetal bovine serum (FBS) and 1% penicillin − streptomycin at 37°C in a 5% CO2 incubator. The cells were digested and seeded in 6-well plates or dishes, and then grew to ∼70–80% confluence for subsequent experiments.

The cells were treated with insulin (Sigma-Aldrich, I2643, MO, United States) (0, 25, 50, 100, 150, and 200 nM) in DMEM supplemented with free or 10% FBS for 24 h to determine cell proliferation. The cells were treated with insulin (50, 100, and 200 nM) in DMEM supplemented with 10% FBS for 24 h to extract the total RNA. The cells were treated with insulin (100 nM) in DMEM supplemented with 2% FBS for 24 h to determine the proinflammatory cytokines concentrations in culture medium. The cells were treated with or without insulin (100 nM) in DMEM supplemented with 10% FBS for 24 h for other *in vitro* experiments. Microglia can be stimulated by lipopolysaccharide (LPS) (*E. coli*, scrotype 055:B5, Sigma, MO, United States) to M1 phenotype. Cells (primary microglia or BV2 cells) treated with LPS (1 μg/ml) for 24 h were used as positive controls for M1 inflammatory status.

### Enzymes linked immunosorbent assay

Proinflammatory cytokines tumor necrosis factor α (TNFα) and interleukin-1β (IL-1β) concentrations in serum and cell culture medium, insulin concentration in serum and cerebrospinal fluid (CSF), and intracellular ATP content of microglia were determined by using mouse TNFα and IL-1β ELISA kit (Peprotech, Rehovot, Israel), mouse insulin ELISA kit (Jonln Bio, Shanghai, China) and mouse ATP assay kit (Jonln Bio, Shanghai, China) according to the manufacturers’ protocols.

### Cell proliferation assays

BV2 cells (10^4^/well) were cultured in 96-well plates. On the second day when the cells grew to 70–80% confluence, different doses of insulin treatments with or without FBS were performed for 24 h. Then cell proliferation condition was assessed by using cell counting kit-8 (CCK-8) (KeyGEN Biotech, Nanjing, China). Each experimental point contained 6 repeats and at least three biological repeats.

### Western blot analysis

Protein isolation and western blot analysis were followed by the standard protocol as we previously described (Liu et al., 2020). Total protein from brain tissues and cultured cells were homogenized in RIPA lysis buffer (KeyGEN Biotech, Nanjing, China) with protease inhibitor cocktail and phosphatase inhibitor cocktail (Merck, MO, United States). And membrane protein and cytoplasmic protein from cultured cells homogenate were isolated by using a Membrane Protein and Cytoplasmic Protein Extraction Kit (KeyGEN Biotech, Nanjing, China). Proteins were separated by SDS-PAGE eletrophoresis and transferred to a nitrocellulose (NC) filter membrane. NC membranes were blocked with 1% bovine serum albumin (BSA) for 1 h and incubated overnight at 4°C with the primary antibodies: anti-Iba-1 (1:1000, Wako, Saitama, Japan), anti-GLUT4 (1:1000, Proteintech, Wuhan, China), anti-MFN2 (1:1000, Cell Signaling, MA, United States), anti-DRP1 (1:1000, Cell Signaling, MA, United States), anti-phospho- DRP1 (Ser616) (1:1000, Cell Signaling, MA, United States), anti-ATPase Na^+^/K^+^ transporting subunit alpha 1 (ATP1A1) (1:500, Proteintech, Wuhan, China), anti-phospho-Insulin Receptor (InsR) β (Tyr1361) (1:1000, Cell Signaling, MA, United States), anti-InsR β (1:1000, Cell Signaling, MA, United States), anti-Akt (1:1000, Cell Signaling, MA, United States), anti-phospho-Akt (Ser473) (1:1000, Cell Signaling, MA, United States), and anti-glyceraldehyde-3-phosphate dehydrogenase (GAPDH) (1:5000, Proteintech, Wuhan, China). NC membranes were incubated with fluorescence labeling secondary antibody (1:15000; LI-COR, Lincoln, NE, United States) for 60 min at room temperature. Quantitative densitometry was performed and analyzed on the protein bands by using Odyssey CLx Imager and Image Studio software (LI-COR, NE, United States).

### Quantitative real-time PCR

Total RNA was extracted from the brain tissue and cultured cells by Trizol reagent (Takara, Osaka, Japan) according to the protocol as we previously described (Zhao et al., 2021). The concentration of RNA was quantified by ultraviolet spectrophotometry at 260/280 nm (Nanodrop 2000, Thermo scientific, MA, United States). The HiScript II Q RT SuperMix for qPCR (+gDNA wiper) (Vazyme, Nanjing, China) was used to transcribe cDNA. qRT-PCR was performed on a Mx3000P (Bio-rad, CA, United States) quantitative real-time detection system with corresponding primers and ChamQ Universal SYBR qPCR Master Mix (Vazyme, Nanjing, China). The thermal cycling parameters of qRT-PCR includes 95°C 30 s, 1 cycle; 95°C 10 s, 60°C 30 s, 40 cycles. The normalized expressions of targeted mRNA were calculated by the comparative CT method, and β-actin was used as the internal standard reference. The fold changes were calculated by using the 2^–ΔΔ*Ct*^ method (Liu et al., 2019). The primer sequences (synthesized by Thermo scientific, MA, United States) are the following:

tnf-α forward: 5′ATCCGCGACGTGGAACTG 3′

tnf-α reverse: 5′ACCGCCTGGAGTTCTGGAA 3′

il-1β forward: 5′GAGCACCTTCTTTTCCTTCATCTT 3′

il-1β reverse: 5′TCACACACCAGCAGGTTATCATC 3′

cd86 forward: 5′AGCACGGACTTGAACAACCAGAC 3′

cd86 reverse: 5′TTGTAAATGGGCACGGCAGATA 3′

il-6 forward: 5′CAACGATGATGCACTTGCAGA 3′

il-6 reverse: 5′CTCCAGGTAGCTATGGTACTCCAGA 3′

β-actin forward: 5′TCATCACTATTGGCAACGACG 3′

β-actin reverse: 5′AACAGTCCGCCTAGAAGCAC 3′

### Statistical analysis

**FIGURE 1:**
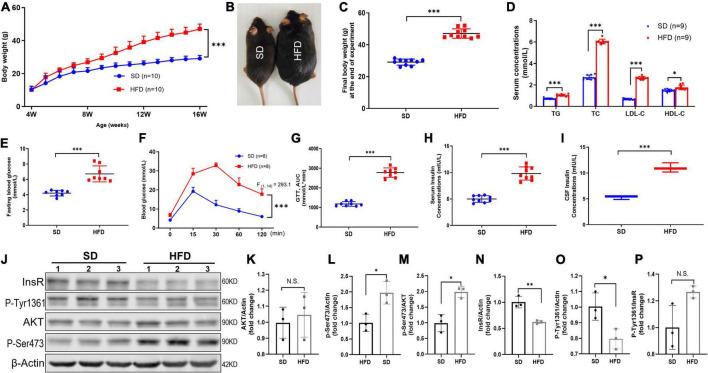
HFD led to increased body weight, disorder of systemic glucose/lipid metabolism, hyperinsulinemia, and brain IR in mice. **(A)** Body weight changed of mice fed with SD and HFD. *n* = 9 per group. **(B)** Representative photographs of the SD and HFD feeding mice at the end of experiment. **(C)** The final body weight at the end of experiment in SD and HFD feeding mice. **(D)** The circulating lipids levels in the SD and HFD groups. *n* = 9 per group. **(E)** The fasting blood glucose average levels in the SD and HFD feeding mice. *n* = 8 per group. **(F)** Blood glucose concentration at different time points after glucose intraperitoneal injection in GTT. *n* = 8 per group. **(G)** AUC values of GTT were calculated and compared between SD and HFD group mice. *n* = 8 per group. **(H)** Serum insulin concentrations in SD and HFD group. *n* = 9 per group. **(I)** CSF insulin concentrations in SD and HFD group. *n* = 9 per group. **(J)** Representative blot images of Western blot for total AKT, p-AKT (Ser473), InsR, and p-InsR (Tyr1361) in mice whole brains, β-Actin served as an equal loading control. **(K–P)** The corresponding densitometry of total AKT **(K)**, p-AKT Ser473 **(L)**, p-S473/total AKT **(M)**, total InsR **(N)**, p-InsR Tyr1361 **(O)**, and p-T1361/total InsR **(P)** expression in mice brains (fold change of control group). *n* = 3 per group. Data are expressed as mean ± SD. *p < 0.05, **p < 0.01, ***p < 0.001.

**FIGURE 2:**
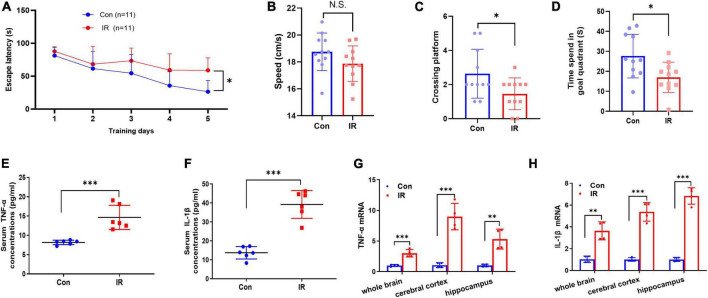
MWM test and proinflammatory factors expression in serum and brain. **(A–D)** The results of MWM test, *n* = 11 per group. **(A)** The escape latency to reach the platform during navigation training session on different day. **(B)** The swimming speed. **(C)** Platform crossing frequency. **(D)** Resident duration in goal quadrant. **(E,F)** The pro-inflammatory cytokines TNFα **(E)** and IL-1β **(F)** concentrations in serum. *n* = 8 per group. **(G,H)** The mRNA expression of pro-inflammatory cytokines TNFα **(G)** and in IL-1β **(H)** in different brain areas. *n* = 3 per group. Data are expressed as mean ± SD. *p < 0.05, **p < 0.01, ***p < 0.001.

Prism 7.0 software (GraphPad Software Inc., CA, United States) was used for statistical analysis. Data were first tested for normality (Shapiro–Wilk test) and the appropriate statistical tests were chosen. Data are expressed as mean ± SD. The significance level was set at *P* < 0.05. The unpaired two-tailed Student’s *t*-test was used to analyze the data sets with a single independent variable factor. Two-way ANOVA was used among these more than groups, followed by Tukey’s *post-hoc* test for multiple comparisons test (Figures 4A–M). Body weight, blood glucose curve in GTT and learning curves of the MWM test data were assessed by repeated-measures ANOVA, as shown in Figures 1A,F, 2A.

## Results

### Establishment of prolonged high-fat diet induced insulin resistance mouse model with hyperinsulinemia

Obesity is the most common cause for IR and hyperinsulinemia in mammals (Eckel et al., 2005; Lauterbach and Wunderlich, 2017). Consequently, we fed 4-week-old mice with HFD for 12 weeks and monitored the body weight every week. The repeated-measures ANOVA demonstrated a significant difference between-subjects effects (for time) in the body weight [*F*_(12_,_216)_ = 755.4, *p* < 0.001] (Figure 1A), suggesting mice gain weight over time. Repeated-measures ANOVA showed a significant difference between-subjects effects (for group) in the body weight [*F*_(1_,_18)_ = 84.89, *p* < 0.001] (Figure 1A), suggesting the body weight of HFD feeding mice increased rapidly compared with the SD mice. At the end of 12 weeks, the body weight of HFD feeding mice was approximately 55% heavier (Figures 1B,C). Meanwhile, HFD feeding mice exhibited obvious disorder of glucose and lipid metabolism, including higher levels of fasting plasma glucose (Figure 1E), AUC of the oral GTT (Figures 1F,G), plasma TG, TC, LDL-C, and HDL-C (Figure 1D). Moreover, significantly increased insulin level was found in both serum (Figure 1H) and CSF (Figure 1I) in HFD feeding mice, suggesting prolonged HFD led to IR characterized by hyperinsulinemia and glucose metabolic disorders.

Furthermore, other studies have reported that obesity also induces brain IR (Spinelli et al., 2017). To further evaluate the HFD-induced brain IR, we investigated the insulin signaling pathway by Western blot. Indeed, HFD significantly decreased the total expression of InsR β, and increased the basal phosphorylation of Akt at Ser473 (p-Akt S473) in the whole brains. But HFD did not alter the phosphorylation of InsR at Tyr1361 (p-InsR T1361) level and total AKT expression (Figures 1J–P). Taken together, HFD also induced whole brain IR.

### Impaired spatial learning and memory function and occurring of inflammation in diet-induced insulin resistance mice

Cognitive decline and neuroinflammation are associated with the earlier onset of AD (Minter et al., 2016; Sharma, 2019), and therefore we further examined and compared the effects of IR on the cognitive function and inflammation. Initially, mice were tested in the MWM task for both learning and reference memory. In the spatial navigation test, the repeated-measures ANOVA demonstrated a significant difference between-subjects effects (for time) in the escape latencies [*F*_(4_,_80)_ = 20.77, *p* < 0.001] (Figure 2A), suggesting intact learning capabilities of these mice. And a significant difference between-subjects effects (for group) in the escape latencies [*F*_(1_,_20)_ = 7.978, *p* = 0.0105] (Figure 2A) suggested a significance between two groups. The escape latency to reach the platform in the IR mice group was significantly longer than that in control mice group, showed decreased learning ability in IR mice. The repeated-measures ANOVA showed no significant difference within-subjects effects (time × group) [*F*_(4_,_80)_ = 2.351, *p* = 0.061] (Figure 2A). In the spatial probe trial, both platform crossing frequency and goal quadrant resident time in IR mice were significantly shorter than those in control mice (Figures 2C,D), although the swimming speed had no significant difference (Figure 2B), indicating that both learning ability and memory of IR mice were impaired.

Substantial evidence suggests that IR- induced neuroinflammation may affect cognitive function, so next we detected the expression levels of pro-inflammatory cytokines TNF-α and IL-1β in both periphery and the different brain regions. The results showed that both TNF-α and IL-1β in serum were significantly increased in the IR mice (Figures 2E,F), and the mRNA expression levels of these two factors also were significantly upregulated in different brain regions including whole brain, cerebral cortex and hippocampus in IR mice (Figures 2G,H). These results demonstrated that cognitive impairment and inflammation in both periphery and the CNS occurred in diet-induce IR mice.

### Prolonged high-fat diet induced microglia activation and M1-like polarization in mice

**FIGURE 3:**
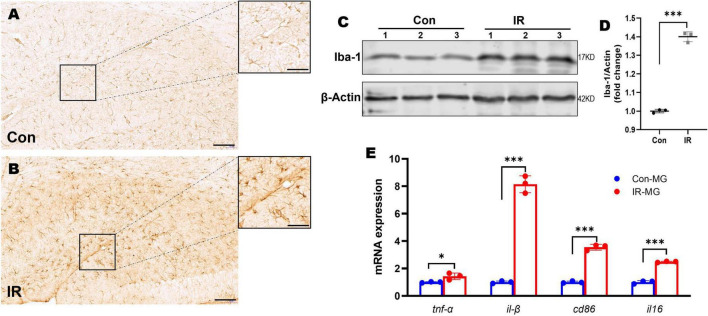
Microglia activation and M1 polarization in HFD-induced IR mice. **(A,B)** Iba-1 IHC staining in hippocampus, panel **(A)** showed control mice, panel **(B)** showed IR mice. *n* = 3 per group. **(C)** Representative blot images of Western blot for Iba-1 protein expression in the whole hippocampus. β-Actin served as an equal loading control. **(D)** The corresponding densitometry of Iba-1 (fold change of control group). *n* = 3 per group. **(E)** The expression of CD86, CD16, TNFα, and IL-1β mRNA in purified microglia from mice brains by qPT-PCR. *n* = 3 per group. Scale bar: 100 μm in panels **(A,B)**, 20 μm in the enlarged box in panels **(A,B)**. Data are expressed as mean ± SD. *p < 0.05, ^***^p < 0.001.

Hippocampus is intimately associated with cognition behaviors (Moser and Moser, 1998). Microglia is the immune cell of the CNS and consequently plays important roles in neuroinflammation (Goldmann and Prinz, 2013). Therefore, we examined the morphological changes of microglia by using Iba1 IHC staining. Compared to the control mice, Iba1 staining density and intensity were strongly increased in the hippocampus of IR mice (Figures 3A,B), and Iba1 positive cells showed retracted processes and increased soma size which implying activated status (in the enlarged box of Figures 3A,B) (Nimmerjahn, 2012; Colonna and Butovsky, 2017). To quantify the overall state of microglia in the hippocampus, Western blot was carried out by using Iba1 as the marker. The results showed that Iba1expression was also significantly elevated in the whole hippocampi in IR mice (Figures 3C,D).

Activated microglia has two distinguish functional status, namely M1 and M2 type microglia (Cao and He, 2013). M1 type microglia can promote neuroinflammation by releasing pro-inflammatory cytokines (Fraser et al., 2010) and involved in the pathogenesis of neurodegeneration disease including AD (Walker and Lue, 2015; Wang et al., 2021). In order to understand the microglia activation status *in vivo*, we obtained single cells suspensions from mice brains by using Adult Brain Dissociation Kit (Miltenyi Biotec), then CD11b MicroBeads, MS Column and MiniMACS Separator (Miltenyi Biotec) were carried out to obtain purified microglia for RNA isolation. The mRNA expression levels of M1 markers including TNFα, IL-1β, CD86, and IL-6, were tested by qPT-PCR. The results showed that M1 markers’ mRNA expression levels were significantly higher in those microglia from IR mice (Figure 3E), suggesting activated microglia polarized toward M1 status in the HFD-induced IR mice.

### Hyperinsulinemia promoted microglia proliferation and M1-like polarization *in vitro*

**FIGURE 4:**
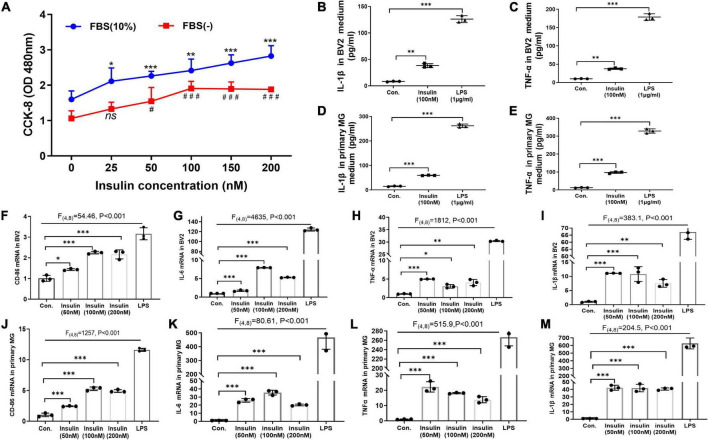
Hyperinsulinemia promoted microglia proliferation and M1-like polarization *in vitro*. **(A)** CCK-8 assay detected the BV2 cell proliferation with different doses of insulin in the free or 10% FBS culture condition. **(B)** The level of pro-inflammatory cytokine IL-1β in BV2 cell culture supernatant. **(C)** The level of pro-inflammatory cytokine TNFα in BV2 cell culture supernatant. **(D)** The level of pro-inflammatory cytokine IL-1β in primary microglia culture supernatant. **(E)** The level of pro-inflammatory cytokine TNFα in primary microglia culture supernatant. **(F–I)** The mRNA expression of M1 marker CD86 **(F)**, IL-6 **(G)**, TNFα **(H)**, and IL-1β **(I)** in BV2 cells after insulin treatments with different doses. **(J–M)** The mRNA expression of M1 marker CD86 **(J)**, IL-6 **(K)**, TNFα **(L)**, and IL-1β **(M)** in primary microglia after insulin treatments with different doses. Cells treated with 1 μg/ml LPS for 24 h were served as positive controls for M1 inflammatory status. Data are expressed as mean ± SD. ^*,#^*p* < 0.05, ^**^p < 0.01, **^*,###^*p* < 0.001. *n* = 3 per group.

So far, we have demonstrated that neuroinflammation and microglia M1-like polarization in HFD-induced IR mice. Next, we asked whether hyperinsulinemia could directly active microglia in an independent manner. We performed insulin treatment in both BV2 cell line and primary cultured microglia for 24 h *in vitro*, to mimic hyperinsulinemia condition *in vivo*. At first, we challenged BV2 cells with different doses of insulin (25, 50, 100, 150, and 200 nM) for 24 h in the free or 10% FBS culture condition, then cell proliferation status was detected, respectively. The two-way ANOVA demonstrated a significant difference between-subjects effects (for FBS) in cell proliferation [*F*_(1,47)_ = 112.3, *P* < 0.001], suggesting FBS promotes cell proliferation (Figure 4A). And a significant difference between-subjects effects (for insulin doses) in cell proliferation [*F*_(5,47)_ = 22.53, *P* < 0.001] suggested insulin itself can promote cell proliferation (Figure 4A). There was no significant interaction between FBS and insulin [*F*_(5,47)_ = 0.9638, *P* = 0.4495]. Tukey’s *post-hoc* test for multiple comparisons was used to compare the proliferation of different doses of insulin treated cells with that of the control group. We found that cell proliferation was in an insulin dose-dependent manner (Figure 4A).

Meanwhile, the effect of different doses of high levels insulin (50, 100, and 200 nM) on M1 polarization of microglia was detected by qRT-PCR. LPS-treated cells were used as positive controls. The two-way ANOVA and Tukey’s *post-hoc* test for multiple comparisons demonstrated high levels insulin increased the mRNA expression of M1 markers (TNFα, IL-1β, CD86, and IL6) in both BV2 cells (Figures 4F–I) and primary microglia (Figures 4J–M), although there was no obvious dose dependence.

Furthermore, by using ELISA we analyzed the released pro-inflammatory factors in the culture supernatant of BV2 cells and primary microglia after insulin (100 nM) treatment. Significant differences of IL-1β [BV2: *F*_(2_,_4)_ = 556.5, *p* < 0.001; primary microglia: *F*_(2_,_4)_ = 2794, *p* < 0.001] and TNFα [BV2: *F*_(2_,_4)_ = 807.1, *p* < 0.001; primary microglia: *F*_(2_,_4)_ = 1604, *p* < 0.001] expression are shown in both BV2 cells (Figures 4B,C) and primary microglia (Figures 4D,E) among these groups by two-way ANOVA. Tukey’s *post-hoc* test for multiple comparisons showed that the insulin group exhibited significantly increased IL-1β and TNFα expression compared to the control group (Figures 4B–E), implying hyperinsulinemia could promote the production and release of pro-inflammatory cytokines which might be caused by insulin-mediated microglia proliferation and M1-like polarization.

### Hyperinsulinemia affected microglia energy metabolism and mitochondrial function *in vitro*

**FIGURE 5:**
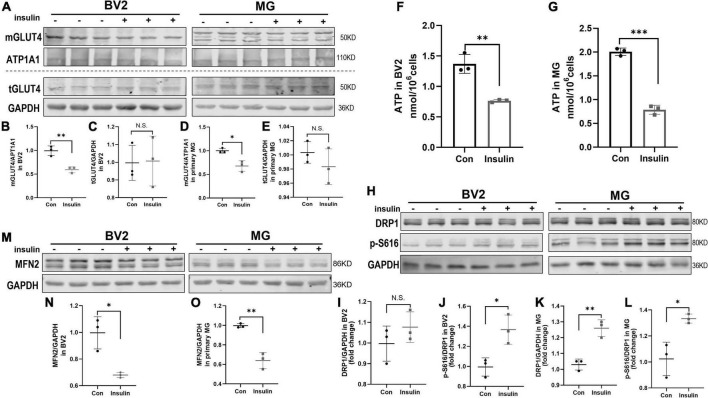
GLUT4 expression, mitochondrial dynamic, and ATP content in BV2 cells and primary microglia after insulin treatment *in vitro*. **(A–E)** Membranous and total GLUT4 expressions in BV2 cells and primary microglia. **(A)** Representative blot images for membranous and total GLUT4 expression in cells. ATP1A1 and GAPDH served as equal loading controls for membranous and total protein, respectively. **(B–E)** The corresponding densitometry of membranous and total GLUT4 expression in BV2 and primary microglia cells (fold change of control group). **(F)** Intracellular ATP content in BV2 cells. **(G)** Intracellular ATP content in primary microglia. **(H)** Representative blot images of Western blot for total DRP1 and p-DRP1 S616 in BV2 cells and primary microglia, GAPDH served as an equal loading control. **(I–L)** The corresponding densitometry of total and p-S616/total DRP1 expression in BV2 cells and primary microglia (fold change of control group). **(M)** Representative blot images of Western blot for MFN2 in BV2 cells and primary microglia, GAPDH served as an equal loading control. **(N,O)** The corresponding densitometry of MFN2 expression in BV2 cells and primary microglia (fold change of control group). Data are expressed as mean ± SD. *p < 0.05, ^**^p < 0.01, ^***^p < 0.001. *n* = 3 per group.

It has been widely recognized and valued that altered metabolic function can affect the microglia activation status (Orihuela et al., 2016; Zhao et al., 2017). Insulin is the critical hormone in energy metabolism by regulating cellular uptake of glucose. GLUT4 is the insulin-responding glucose transporter which promotes glucose uptake through its membrane translocation (Karnieli et al., 1981). GLUT4 has been found in brain in specific region including hippocampus and also expressed by microglia (Nijland et al., 2014; Koepsell, 2020). However, the alteration of GLUT4 expression in microglia undergoing hyperinsulinemia has not been known although its defects are observed in IR and metabolic syndrome (McNay and Pearson-Leary, 2020; Yu et al., 2022). In present study, we analyzed membranous and total GLUT4 expression in insulin- treated BV2 cells and primary microglia which in M1 active status by Western blot. The results showed that insulin decreased the membranous expression of GLUT4 but did not alter its total expression both in BV2 cells and primary microglia (Figures 5A–E), suggesting hyperinsulinemia impaired GLUT4 membrane translocation in microglia. Meanwhile, we detected the production of ATP in the cells in the same condition. We found that ATP production was significantly downregulated both in the BV2 cells (Figure 5F) and primary microglia (Figure 5G), indicating hyperinsulinemia resulted in dysfunction of energy metabolism in microglia.

Adenosine triphosphate is commonly produced in mitochondria which are highly dynamic organelles undergoing coordinated cycles of fission and fusion, namely mitochondrial dynamics (Hatefi, 1993; Chan, 2020). Mitochondria transient and rapid morphological adaptations are crucial for many cellular processes, such as cell cycle, immunity, apoptosis, and mitochondrial quality control (Buck et al., 2016; Horbay and Bilyy, 2016; Rivers et al., 2019; Ma et al., 2021). On the other hand, mitochondrial dynamics can also reflect their functional state. In this study, we used phosphorylated DRP1 at Ser616 (p-DRP1 S616) and MFN2 as the marker of fission and fusion to examine the effects of hyperinsulinemia on the mitochondrial dynamics in microglia by Western blot. The results showed that p-DRP1 S616 was significantly increased (Figures 5H–L), contrary MFN2 expression was decreased (Figures 5M–O) in both BV2 cells and primary microglia treated by insulin, suggesting hyperinsulinemia made mitochondrial fission and fusion out of balance in microglia.

## Discussion

This study was designed to investigate the effects of hyperinsulinemia on the microglia function, metabolism and its related neuroinflammation. We found that long-term HFD successfully provoked mice IR combined with peripheral and CNS hyperinsulinemia, brain IR, exhibited neuroinflammation, and spatial learning memory deficits. Meanwhile, more significant microglia activation in the hippocampus and M1-like polarization in the whole brain were observed in IR mice. Furtherly, *in vitro* studies provided the direct evidence that hyperinsulinemia induced microglia activation and M1-like polarization. And alterations in cellular bioenergetics in insulin-treated microglia *via* mitochondrial dynamics imbalance, diminished GLUT4 membrane translocation, and intracellular ATP content were also identified. All these findings suggested that hyperinsulinemia provoked microglia activation and M1 polarization by interfering with metabolism, which might be related to the development of neuroinflammation in IR mice.

Epidemiological studies suggest that obesity could significantly and independently increase the risk for AD (Ho et al., 2010; Profenno et al., 2010). So far, the detailed mechanisms linking obesity to AD have been widely discussed and various factors have been suggested, in which IR is a key risk factor for the impact on cognitive functions. Obesity is the commonest cause of secondary IR in human, and diet induced obesity and IR animal models were widely used in the laboratory (Chen et al., 2019; Rivers et al., 2019). Hence, C57BL/6J background male mice (4-week-old) which are susceptible to HFD feeding (Fisher-Wellman et al., 2016) were used in present study to mimic IR condition. The results showed that a 12-week HFD feeding successfully induced multiple features related to IR in mice, including obesity, dysregulated glucose and lipid metabolism, and hyperinsulinemia. Meanwhile, aberrant insulin signaling was found in the brain of HFD mice, consisting of basal hyper-phosphorylation of Akt and hypo-expression of InsR (Figure 1). Since IR has been linked with cognitive disfunction (Kim and Feldman, 2015; Ahmad and Al-Domi, 2018; Wang et al., 2018), we thus identified spatial learning and memory of mice by behavior analysis and found their deficits on cognitive functions in IR mice (Figures 2A–D).

Numerous studies have identified the complex links among IR, AD and neuroinflammation (Cai et al., 2015; Guillemot-Legris and Muccioli, 2017). In our present study, we found activated microglia and M1 markers’ expressions were upregulated in IR mice (Figure 3), implying M1 microglia might be the critical factor involved in IR induced neuroinflammation. Under IR conditions, microglia activation is associated with multiple factors, such as increased fatty acid, disruption of BBB and stimulation of peripheral proinflammatory factors (Lumeng and Saltiel, 2011; Stranahan et al., 2016; Van Dyken and Lacoste, 2018; Yang et al., 2020). However, the role of hyperinsulinemia which is the feature of IR and occurs at the early stage of T2D, on microglial activation is still unknown. Increased evidence suggests that hyperinsulinemia participates in a variety of diseases as an independent factor (Weyer et al., 2000; Hegde et al., 2019; Zhang et al., 2021), and hyperinsulinemia in itself can lead to IR rather than a consequence of IR (Pandey et al., 2015; Lee et al., 2017). In the periphery, a strong association between hyperinsulinemia and chronic low-grade inflammation has been demonstrated, for instance, obesity-associated hyperinsulinemia can drive adipose tissue inflammation (Pedersen et al., 2015); hyperinsulinemia can promote macrophages to produce pro-inflammatory factors (Mauer et al., 2010; Manowsky et al., 2016), and so on. Hence, we speculated hyperinsulinemia had a similar effect on microglia in CNS. As expected, our results indicated hyperinsulinemia could promote microglia proliferation, M1 polarization and pro-inflammatory factors release (Figure 4).

As highly dynamic cells, microglia carry out a non-stop patrol of the CNS, rapidly activate and exhibit either M1 or M2 phenotype to maintain brain homeostasis (Tang and Le, 2016). Accumulating evidence supports that microglia exhibit different metabolic profiles in the face of alterations in the microenvironment, that to support morphological changes and functional remodeling in the process of cell polarization (Orihuela et al., 2016; Luo et al., 2021). Insulin is necessary for metabolism regulation and bioenergy production by regulating cellular glucose transport (Summers et al., 1998). GLUT4 as the insulin-responding glucose transporter has been found expressed in neurons in hippocampus and involved in memory function (Birnbaum, 1989; McNay and Pearson-Leary, 2020; Fazakerley et al., 2022), and its defects are observed in IR and metabolic syndrome (McNay and Pearson-Leary, 2020; Yu et al., 2022). GLUT4 expression in microglia has been detected (Nijland et al., 2014; Koepsell, 2020), however, the alteration undergoing hyperinsulinemia has not been known. Our *in vitro* data showed that insulin treatment decreased GLUT4 localized on the membrane of microglia. Meanwhile, decreased intracellular ATP content was identified, indicating the alterations of GLUT4 translocation caused by hyperinsulinemia may affect intracellular energy production through decreased glucose up taking.

Mitochondrial dynamics is a crucial mechanism in adaptation to cell energy requirements and maintains the mitochondrial homeostasis (Ni et al., 2015). And exciting evidence has demonstrated reciprocal crosstalk between mitochondrial dynamics and metabolism. Metabolic cues can control the mitochondrial fission and fusion machinery to acquire specific morphologies that shape their activity (Rambold and Pearce, 2018). And increased mitochondrial fission induced by the activation of DRP1 can promote M1 macrophages through increased glycolysis (Xie et al., 2020). Our study revealed that insulin treatment significantly increased the level of p-DRP1 S616 in microglia (Figures 5H–L), which was concomitant with significant inhibition of MFN2 protein expression (Figures 5M–O). All these indicate that hyperinsulinemia leads to an excessive increase in mitochondrial fission in microglia, which might be related to insufficient glucose intake caused by impaired GLUT4 translocation, and therefore promotes microglial M1 polarization.

In summary, our study demonstrated that IR related hyperinsulinemia promoted microglia M1 polarization and neuroinflammation in the CNS. Decreased GLUT4 membrane translocation, energy metabolism disorder and mitochondrial dynamics imbalance might be involved in hyperinsulinemia induced M1 microglia polarization and neuroinflammation during IR. Our study provides new perspective and evidence for the mechanism underlying the association between T2D and AD.

## Data availability statement

The original contributions presented in this study are included in the article/supplementary material, further inquiries can be directed to the corresponding author.

## Ethics statement

This animal study was reviewed and approved by the Ethics Committee of Dalian Medical University.

## Author contributions

XY and JM designed the study and drafted the manuscript. YX, WG, RD, LW, HH, and GL conducted animal experiments. XZ, SL, and SQ performed the statistical analysis. KF and XM revised the manuscript. All authors contributed to the manuscript and approved the submitted version.

## Abbreviations

ATP: adenosine triphosphate
AD: Alzheimer’s disease
ANOVA: analysis of variance
AUC: area under the curve
ATP1A1: ATPase Na^+^/K^+^ transporting subunit alpha 1
BBB: blood–brain barrier
BSA: bovine serum albumin
CCK-8: cell counting kit-8
CNS: central nervous system
CSF: cerebrospinal fluid
CD11b: cluster of differentiation 11b
CD86: cluster of differentiation 86
DRP1: dynamin-related protein 1
DMEM: Dulbecco’s modified Eagle’s medium
ELISA: enzymes linked immunosorbent assay
FBS: fetal bovine serum
GTT: glucose tolerance tests
GLUT4: glucose transporter 4
GAPDH: glyceraldehyde-3-phosphate dehydrogenase
HDL: high-density lipoprotein
HFD: high-fat diet
IHC: immunohistochemistry
IR: insulin resistance
IL-1β: interleukin-1β
IL-6: interleukin-6
Iba1: ionized calcium binding adapter molecule 1
LPS: lipopolysaccharide
LDL: low-density lipoprotein
MFN2: Mitofusin 2
MWM: Morris water maze
NC: nitrocellulose
PBS: phosphate buffered saline
PAGE: polyacrylamide gel electrophoresis
qRT-PCR: quantitative real-time PCR
SDS: sodium dodecyl sulfate
SPF: specific pathogen free
SD: standard diet
TC: total cholesterol
TG: triacylglycerol
T2D: type 2 diabetes
TNFα: tumor necrosis factor α.
